# Chiral Lattice Resonances in 2.5-Dimensional Periodic Arrays with Achiral Unit Cells

**DOI:** 10.1021/acsphotonics.3c00369

**Published:** 2023-05-10

**Authors:** Luis Cerdán, Lauren Zundel, Alejandro Manjavacas

**Affiliations:** † Instituto de Óptica (IO−CSIC), Consejo Superior de Investigaciones Científicas, 28006 Madrid, Spain; ‡ Department of Physics and Astronomy, University of New Mexico, Albuquerque, New Mexico 87106, United States

**Keywords:** Chirality, Lattice resonances, Periodic arrays, Nanoparticle arrays, 2.5-Dimension, Complex unit cell

## Abstract

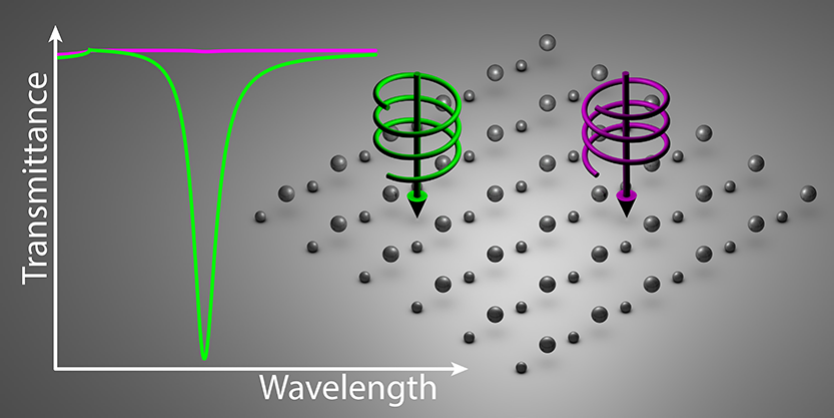

Lattice resonances are collective electromagnetic modes supported by periodic arrays of metallic nanostructures. These excitations arise from the coherent multiple scattering between the elements of the array and, thanks to their collective origin, produce very strong and spectrally narrow optical responses. In recent years, there has been significant effort dedicated to characterizing the lattice resonances supported by arrays built from complex unit cells containing multiple nanostructures. Simultaneously, periodic arrays with chiral unit cells, made of either an individual nanostructure with a chiral morphology or a group of nanostructures placed in a chiral arrangement, have been shown to exhibit lattice resonances with different responses to right- and left-handed circularly polarized light. Motivated by this, here, we investigate the lattice resonances supported by square bipartite arrays in which the relative positions of the nanostructures can vary in all three spatial dimensions, effectively functioning as 2.5-dimensional arrays. We find that these systems can support lattice resonances with almost perfect chiral responses and very large quality factors, despite the achirality of the unit cell. Furthermore, we show that the chiral response of the lattice resonances originates from the constructive and destructive interference between the electric and magnetic dipoles induced in the two nanostructures of the unit cell. Our results serve to establish a theoretical framework to describe the optical response of 2.5-dimensional arrays and provide an approach to obtain chiral lattice resonances in periodic arrays with achiral unit cells.

## Introduction

Periodic arrays of metallic nanostructures support collective electromagnetic modes commonly known as lattice resonances.^1−3^ These modes, which appear in the spectrum at wavelengths commensurate with the periodicity of the array, are the result of the coherent multiple scattering between the individual constituents.^4,5^ Due to their collective nature, lattice resonances give rise to very narrow optical responses,^6−9^ with extraordinarily high quality factors for systems made of metallic nanostructures,^10−13^ and couple strongly with light,^14−16^ producing values of reflectance and absorbance that can reach the theoretical limits for two-dimensional systems.^17,18^ Such exceptional properties have made lattice resonances the subject of an extensive research effort, which has resulted in the proposal and development of a wide range of applications. These include, among others, ultrasensitive biosensors,^19−21^ color filters,^22,23^ light-emitting devices,^24−28^ light-to-heat transducers,^29,30^ and even platforms to explore new physical phenomena.^31−36^

More recently, lattice resonances have begun to be explored for applications involving chirality.^37−40^ A material object is said to be chiral when it cannot be superimposed on its mirror image, or enantiomer, by rotations and translations.^41^ Many important biomolecules, such as sugars and amino acids, as well as drugs used in medicine, are chiral^42,43^ and, in many cases, only one enantiomer is active while the other is inactive or even harmful.^44,45^ Therefore, the precise enantioselective detection, identification, and manipulation of chiral molecules with light-based technologies is a key goal in biosensing and photochemistry, which requires the development of optical systems with strong chiral responses.^46−52^ These systems are also very relevant for the implementation of optical elements capable of controlling and modifying the polarization state of light.^53−55^

The prospects of these applications have triggered the study and characterization of a variety of periodic arrays supporting chiral lattice resonances.^56−64^ The majority of these arrays are built from the repetition of chiral unit cells, made of either individual nanostructures with complex chiral morphologies,^56−58,63^ such as helices,^60^ or multiple nanostructures forming a chiral arrangement.^65,66^ As expected, the lattice resonances supported by these systems exhibit different responses under illumination with right- and left-handed circularly polarized light. More recently, it has been shown that the combination of a rectangular lattice with an achiral unit cell results in arrays capable of supporting lattice resonances with some degree of chirality, which can be enhanced by positioning the array in an inhomogeneous dielectric environment.^59^ Other alternatives that have been explored to obtain chiral lattice resonances in arrays with achiral unit cells involve the use of higher-order lattice resonances^67^ or the introduction of extrinsic chirality^68,69^ through the excitation of the system with tilted illumination.^70−72^

Motivated by these developments, here, we present a different approach to obtain chiral lattice resonances in periodic arrays with achiral unit cells, which is based on the use of 2.5-dimensional arrays. Using a rigorous coupled dipole model, we investigate the lattice resonances supported by square bipartite arrays made of unit cells in which the metallic nanostructures are placed at arbitrary positions in all three spatial dimensions. We label these systems as 2.5-dimensional arrays due to the combination of the two-dimensional nature of the lattice and the three-dimensional geometry of the unit cell. Through a comprehensive analysis of their optical response, we show that these systems can support lattice resonances with almost perfect chiral responses and quality factors exceeding 10^4^ for normal incidence excitation, despite being made of completely achiral unit cells containing two spherical nanostructures. We explain this behavior as the result of the constructive and destructive interference between the different components of the electric and magnetic dipoles induced in the nanostructures of the unit cell. The results of this work set the foundations to investigate the optical response of 2.5-dimensional arrays and provide the fundamental insight necessary to achieve lattice resonances with strong chiral responses using periodic arrays made of achiral unit cells.

## Results and Discussion

**Figure 1:**
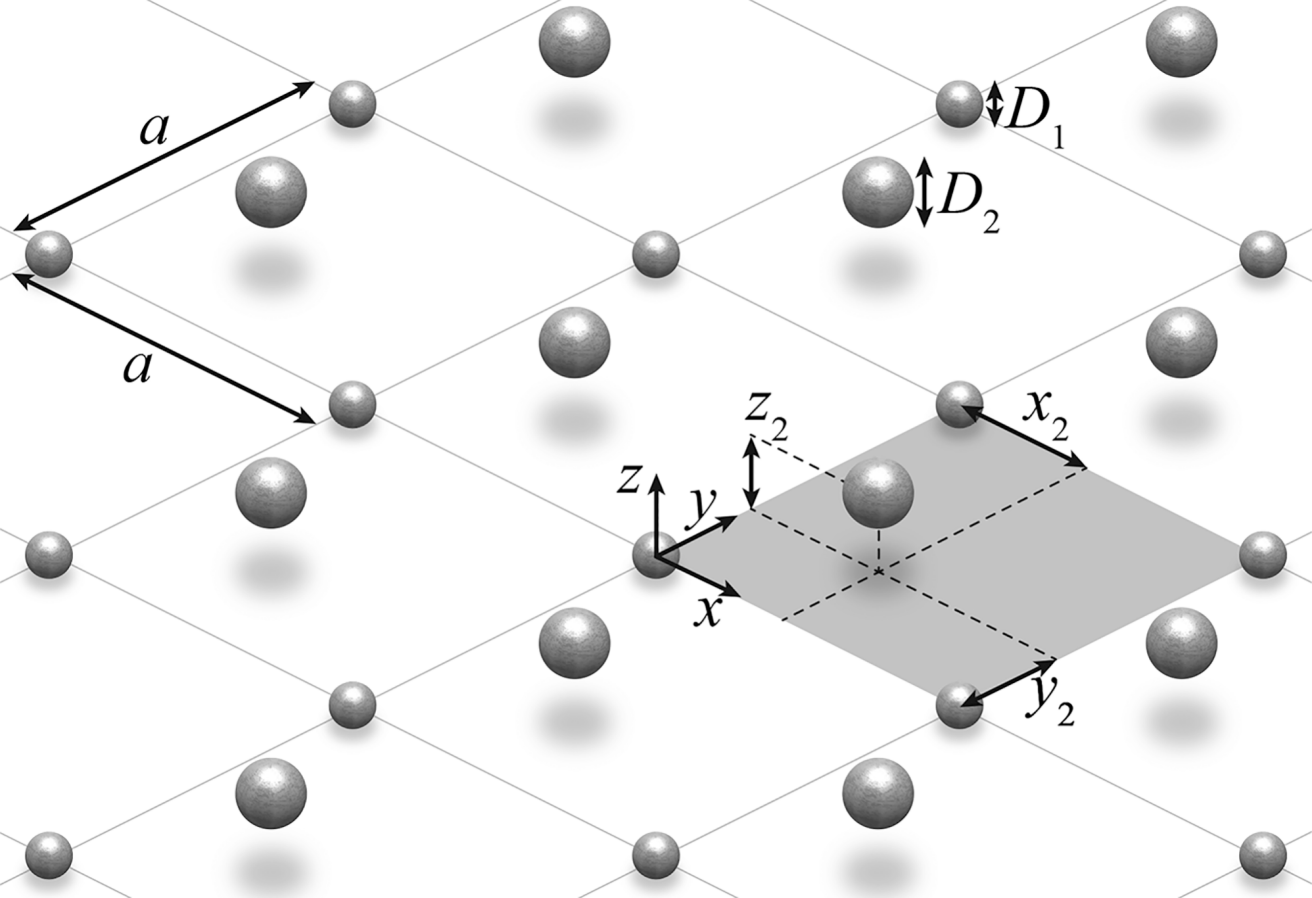
Schematics of the array under study, built from the periodic repetition of a unit cell containing two silver nanospheres (shaded area) over a square lattice with period *a*. The nanospheres have diameters *D*_1_ and *D*_2_, respectively, and are located at positions **r**_1_ = (0, 0, 0) and **r**_2_ = (*x*_2_, *y*_2_, *z*_2_) with respect to the origin of the unit cell. The array is placed in the *xy*-plane, surrounded by vacuum, and excited by a circularly polarized electromagnetic plane wave with wavelength λ that propagates along the *z*-axis.

The system under consideration is depicted in Figure 1. It consists of an infinite square array of period *a* with a unit cell composed of two nanospheres. Particle 1 has diameter *D*_1_ and is located at **r**_1_ = (0, 0, 0), i.e., the origin of the unit cell, while particle 2 has diameter *D*_2_ and is placed at **r**_2_ = (*x*_2_, *y*_2_, *z*_2_). The array lies in the *xy*-plane and is surrounded by vacuum. As explained above, we refer to this array as a 2.5-dimensional system. Unless otherwise stated, we assume arrays made of silver nanoparticles with *a* = 800 nm, *D*_1_ = 120 nm, and *D*_2_ = 190 nm throughout the manuscript. We describe the dielectric function of silver using the tabulated data compiled in ref (73). To investigate the chiral properties of the optical response of the array, we excite it with either right-handed circularly polarized (RCP) or left-handed circularly polarized (LCP) electromagnetic plane waves with wavelength λ that propagate along the *z*-axis. Notice that we use Gaussian units and define the handedness of the circularly polarized light such that the electric **E** and magnetic **H** fields satisfy **H** = −*i***E** for RCP and **H** = *i***E** for LCP.

It is important to remark that, in contrast to previous works, we consider a square array to maximize the symmetry of the unit cell.^59^ Furthermore, a unit cell composed of two spherical nanoparticles is both intrinsically and extrinsically achiral regardless of their relative position and size.^74^ This can be understood by realizing that the two nanospheres, together with the wavevector of the electromagnetic plane wave that excites them, define a plane. Therefore, the mirror image of such a system can always be superimposed to the original by performing a rotation.^75^ Accordingly, any chiral response displayed by the 2.5-dimensional array under consideration must arise from the collective electromagnetic interactions between its constituents.

We are interested in the optical response associated with the lowest-order lattice resonance of the array, which appears in the spectrum at wavelengths slightly larger than the first Rayleigh anomaly, i.e., at λ ≳ *a*. Importantly, the size of the nanoparticles forming the arrays under consideration are significantly smaller than *a*. Consequently, their localized plasmons are spectrally located far from the lattice resonances, thus ensuring that the latter are highly collective.^30,76^ Furthermore, under these conditions, we can describe the optical response of the array using the well-established coupled dipole model (CDM).^4,5,9,77−80^ Within this semianalytical approach, each nanoparticle is modeled as a point dipole with both electric and magnetic components, and the collective response of the array arises from the self-consistent mutual interaction between the dipoles. To capture the rich phenomenology expected for the arrays under study, we need to take into account the magnetic response of the particles^36,79^ as well as the multipartite and three-dimensional nature of the unit cell.^4,9,13,81^ The resulting approach, which is explained in detail in the Methods, is in excellent agreement with full numerical solutions of Maxwell’s equations obtained using a finite element method (FEM) solver, as demonstrated in Figure S1 of the Supporting Information. However, in contrast to the full numerical solutions, the CDM provides the necessary insight to understand the origin of the optical response of the array.

Once the induced dipoles are computed, we calculate the transmittance *T*, reflectance *R*, and absorbance *A* of the array from the balance between the power that it radiates and absorbs and the power that is incident on it (see the Methods). Moreover, to characterize the chiral response of the array, we use the dissymmetry factor, defined as^59,60^
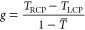
where *T*_RCP_ and *T*_LCP_ are the transmittances for RCP and LCP excitation, respectively, and *T̅* is their average. This quantity, which is inspired by Kuhn’s dissymmetry factor (or *g*-factor) used in spectroscopy,^82^ quantifies the circular dichroism of the array. Notice that, by construction, −2 ≤ *g* ≤ 2, while the choice of sign is arbitrary and bears no physical meaning.

**Figure 2:**
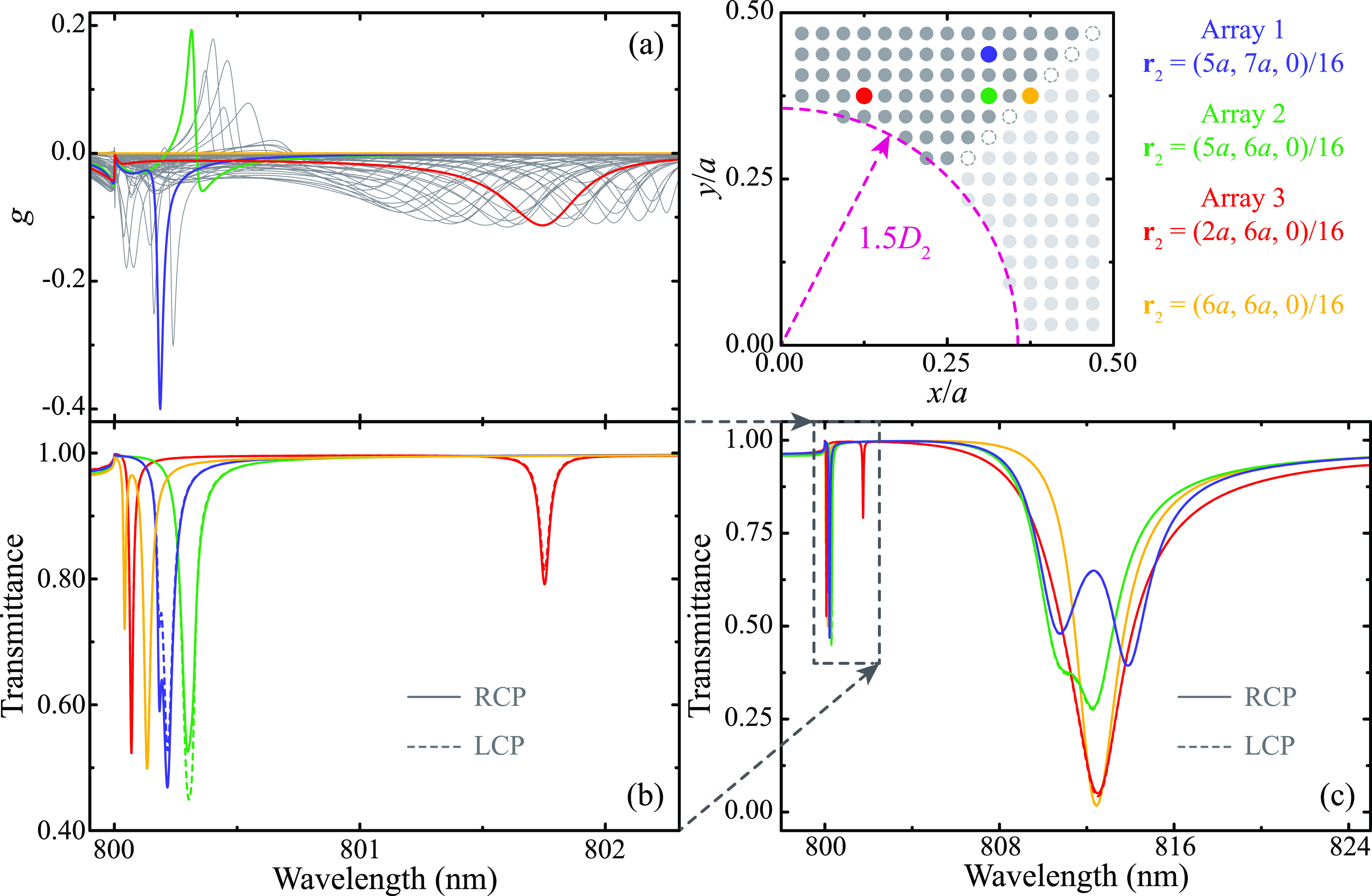
Chiral response of planar arrays. (a) Spectra of the dissymmetry factor for two-dimensional square bipartite arrays in which particle 2 is located at the different positions marked in the accompanying diagram of the lower left quadrant of the unit cell. (b) Spectra of the transmittance under RCP (solid lines) and LCP (dashed lines) excitation for the four selected positions. (c) Zoom-out of panel (b). The color code in all panels matches the one used in the diagram of the quadrant of the unit cell. For all of the arrays, *a* = 800 nm, *D*_1_ = 120 nm, and *D*_2_ = 190 nm.

We begin our study by analyzing the chiral response of conventional two-dimensional square bipartite arrays in which particle 2 is located in the *xy*-plane. Figure 2(a) shows the value of the dissymmetry factor *g* as a function of wavelength for different positions of particle 2 within the unit cell. Specifically, we consider the positions signaled with blue, green, red, and dark gray dots in the diagram located to the right of Figure 2(a). These positions span the upper bisector of the lower left quadrant of the unit cell and fulfill the condition |**r**_1_ – **r**_2_| > 1.5*D*_2_, so that the dipole approximation is sufficiently accurate. Furthermore, given the square symmetry of the array, they are representative of the entire unit cell. For instance, the positions marked with light gray dots are equivalent to those under consideration upon a rotation of 180° around the diagonal of the unit cell. Such rotation is tantamount to changing the handedness of the incident light and, hence, each position below the diagonal results in the same *g* but with opposite sign as the equivalent position above it. The same argument holds true for the positions (*x*_2_, *a* – *y*_2_) and (*a* – *x*_2_, *y*_2_) in the other quadrants, which are equivalent to (*x*_2_, *y*_2_) upon a rotation of 180° around the *x*-axis and *y*-axis, respectively. However, positions (*a* – *x*_2_, *a* – *y*_2_) result in the same value and sign of *g* as (*x*_2_, *y*_2_) because they are connected by a 180° rotation around the *z*-axis.

Examining the results displayed in Figure 2(a), we observe that all of the cases under consideration show a significant chiral response despite corresponding to a two-dimensional square array with an achiral unit cell. The resonant features of *g* in the different spectra can be roughly distributed in two different regions: a first group is located at wavelengths right above the first Rayleigh anomaly, i.e., λ ≳ *a* = 800 nm, and a second one at wavelengths around λ ∼ 801.5 nm. To deepen our analysis, we select three examples representative of these groups, which are indicated with blue, green, and red curves, and correspond, respectively, to the positions **r**_2_ = (5*a*, 7*a*, 0)/16, **r**_2_ = (5*a*, 6*a*, 0)/16, and **r**_2_ = (2*a*, 6*a*, 0)/16. For the sake of simplicity, we refer to these three examples as Arrays 1, 2, and 3 (see diagram). Analyzing the transmittance spectra for these arrays, which are shown in Figure 2(b) for RCP (solid curves) and LCP (dashed curves) excitation, we observe that each feature in the spectrum of *g* corresponds to a lattice resonance. However, the converse is not true. For instance, Array 3 (red curve) supports two lattice resonances in the spectral region under consideration, but only one of them shows a significant value of *g*. Overall, the lattice resonances have a relatively moderate strength, with values of transmittance around 0.5, and a quality factor of ∼10^4^. Importantly, all of these arrays support a much stronger and broader lattice resonance at larger wavelengths, as can be seen in the zoomed-out spectra shown in Figure 2(c). However, these modes display a negligible chirality, as demonstrated by the practically identical transmittance spectra obtained under RCP and LCP excitation.

To complete the analysis of the two-dimensional arrays, we also calculate the spectrum of *g* and *T* for an array in which particle 2 is located along the diagonal of the unit cell at **r**_2_ = (6*a*, 6*a*, 0)/16 (see the yellow dot in the diagram). The corresponding results, which are displayed in Figures 2(a)–(c) with a yellow curve, show that, in this case, the array supports several lattice resonances in the spectral regions under consideration, but, as expected from the highly symmetric position of the particles in the unit cell, they have no chiral response. The same results are obtained for other positions located along any of the remaining symmetry axes of the unit cell.

**Figure 3:**
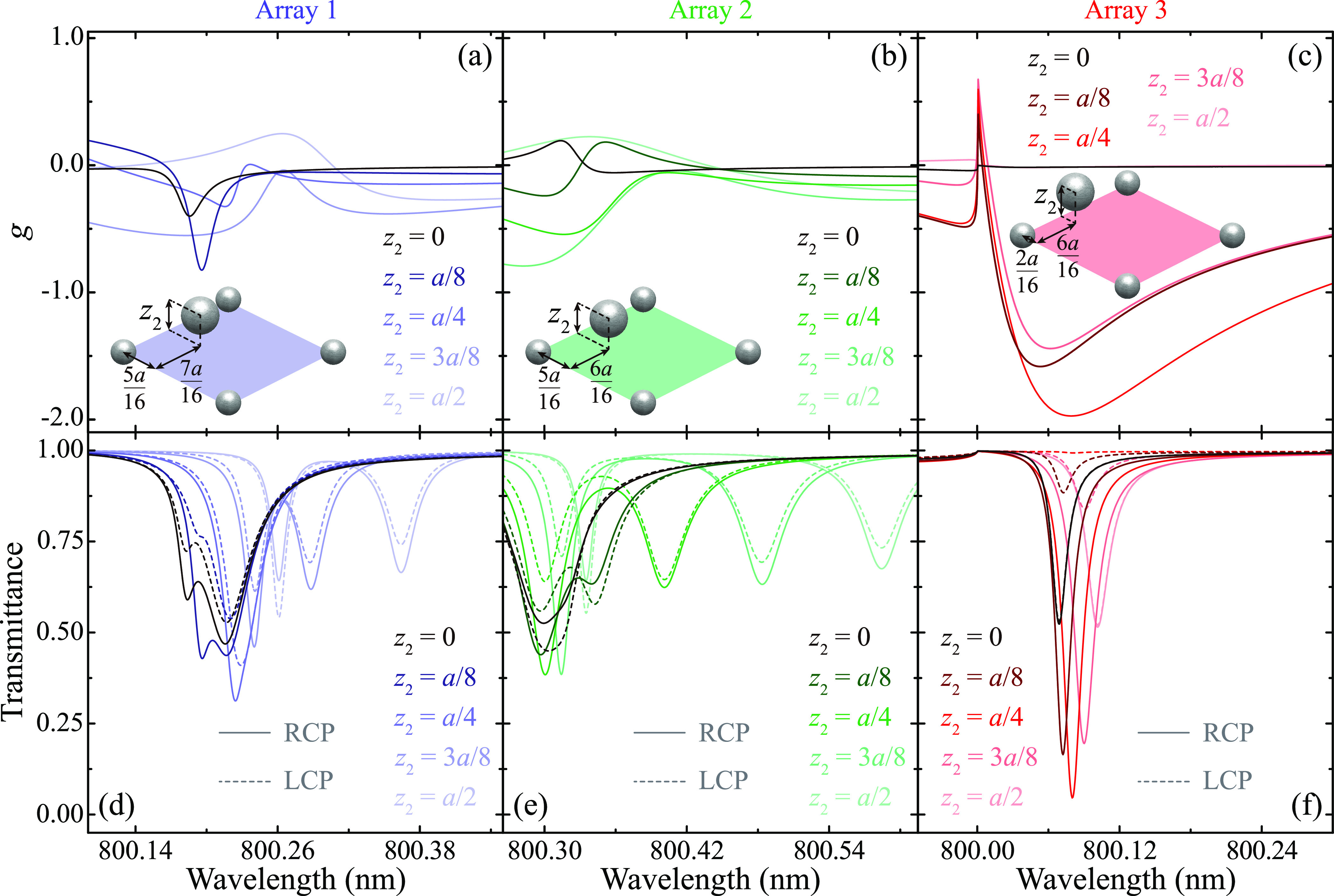
Chiral response of 2.5-dimensional arrays. Dissymmetry factor (a–c) and transmittance (d–f) spectra for three arrays of Figure 2(b) with particle 2 located at different heights *z*_2_ above the *xy*-plane (see legend). Specifically, we consider Array 1 with **r**_2_ = (5*a*, 7*a*, *z*_2_)/16 (a,d), Array 2 with **r**_2_ = (5*a*, 6*a*, *z*_2_)/16 (b,e), and Array 3 with **r**_2_ = (2*a*, 6*a*, *z*_2_)/16 (c,f). In all panels, solid and dashed curves indicate RCP and LCP excitation, respectively.

With this analysis, we have shown that planar bipartite arrays can display a chiral response if the particles in the unit cell are arranged in low-symmetry configurations. However, the lattice resonances supported by these systems only show a moderate level of strength and chirality. A potential way of improving the response of these modes is to further decrease the symmetry of the unit cell by placing particle 2 outside of the *xy*-plane. The resulting array, which has no mirror symmetry with respect to the *xy*-plane, can be considered a 2.5-dimensional system. We explore this strategy in Figure 3, where we analyze the optical response of 2.5-dimensional arrays in which particle 2 is located at different heights *z*_2_ above the *xy*-plane. In particular, we focus on the three representative arrays highlighted in Figure 2(b). These are Array 1 with **r**_2_ = (5*a*, 7*a*, *z*_2_)/16, Array 2 with **r**_2_ = (5*a*, 6*a*, *z*_2_)/16, and Array 3 with **r**_2_ = (2*a*, 6*a*, *z*_2_)/16. Figure 3(a) shows the evolution with *z*_2_ of the dissymmetry factor of Array 1. As anticipated, moving particle 2 out of plane leads to a stronger chiral response, with about a twofold increase of *g* when the particle is displaced from *z*_2_ = 0 to *z*_2_ = *a*/8. However, a further increase of *z*_2_ up to *a*/2 worsens the chiral response, suggesting that there is an optimal height. In addition, the feature in the spectrum of *g* changes its shape, transitioning from a clean Lorentz-like lineshape for *z*_2_ = 0 to more complex and broader lineshapes for *z*_2_ ≠ 0. Array 2, analyzed in Figure 3(b), shows very similar trends, although, in this case, the largest values of *g* are obtained for *z*_2_ = 3*a*/8. These behaviors can be understood by examining the corresponding transmittance spectra, which are plotted in Figures 3(d) and (e) with solid and dashed curves, respectively, for RCP and LCP excitation. In particular, we observe that, for *z*_2_ = 0, both arrays display a pair of overlapping lattice resonances whose separation grows as *z*_2_ is increased. As this happens, the relative values of the transmittance of each of these resonances under RCP and LCP excitation also change, which, in turn, results in the evolution of the lineshape for *g* discussed above. Importantly, the strength of the lattice resonances supported by these two arrays does not improve significantly with respect to the values obtained for *z*_2_ = 0.

The situation is significantly different for Array 3, whose response is analyzed in Figures 3(c) and (f). For *z*_2_ = 0, although this array supports two lattice resonances within the spectral range considered in Figure 2(b), only the resonance located at λ ≈ 801.8 nm displays a chiral response. However, when *z*_2_ increases, the resonance closer to the Rayleigh anomaly also begins to display a chiral response, which becomes maximum for *z*_2_ = *a*/4. For that configuration, the dissymmetry factor reaches a value of *g* = −1.97, i.e., nearly a perfect chiral response. Simultaneously, the transmittance of the array for RCP shows a pronounced dip with a value that drops to *T* ∼ 0.05 at the lattice resonance, while, for LCP, it remains at *T* ∼ 0.99. These results demonstrate that, if properly designed, 2.5-dimensional arrays can support lattice resonances with maximum chiral response. Importantly, as opposed to some previous works, here, the largest value of *g* is reached at the wavelength at which the optical response of the array is the strongest: the peak of the lattice resonance.

As an additional comment, we note that, for the three arrays analyzed in Figure 3, after reaching its maximum value, the value of *g* decreases as *z*_2_ approaches *a*/2. Although it is not shown in Figure 3, increasing *z*_2_ beyond *a*/2 results, first, in a moderate increase of *g* but with a change of its sign, and then in a gradual decrease until it eventually becomes negligible. Furthermore, moving particle 2 in the opposite direction, that is, along the negative *z*-axis, leads to the exact same results discussed above, but with the opposite sign of *g*.

**Figure 4:**
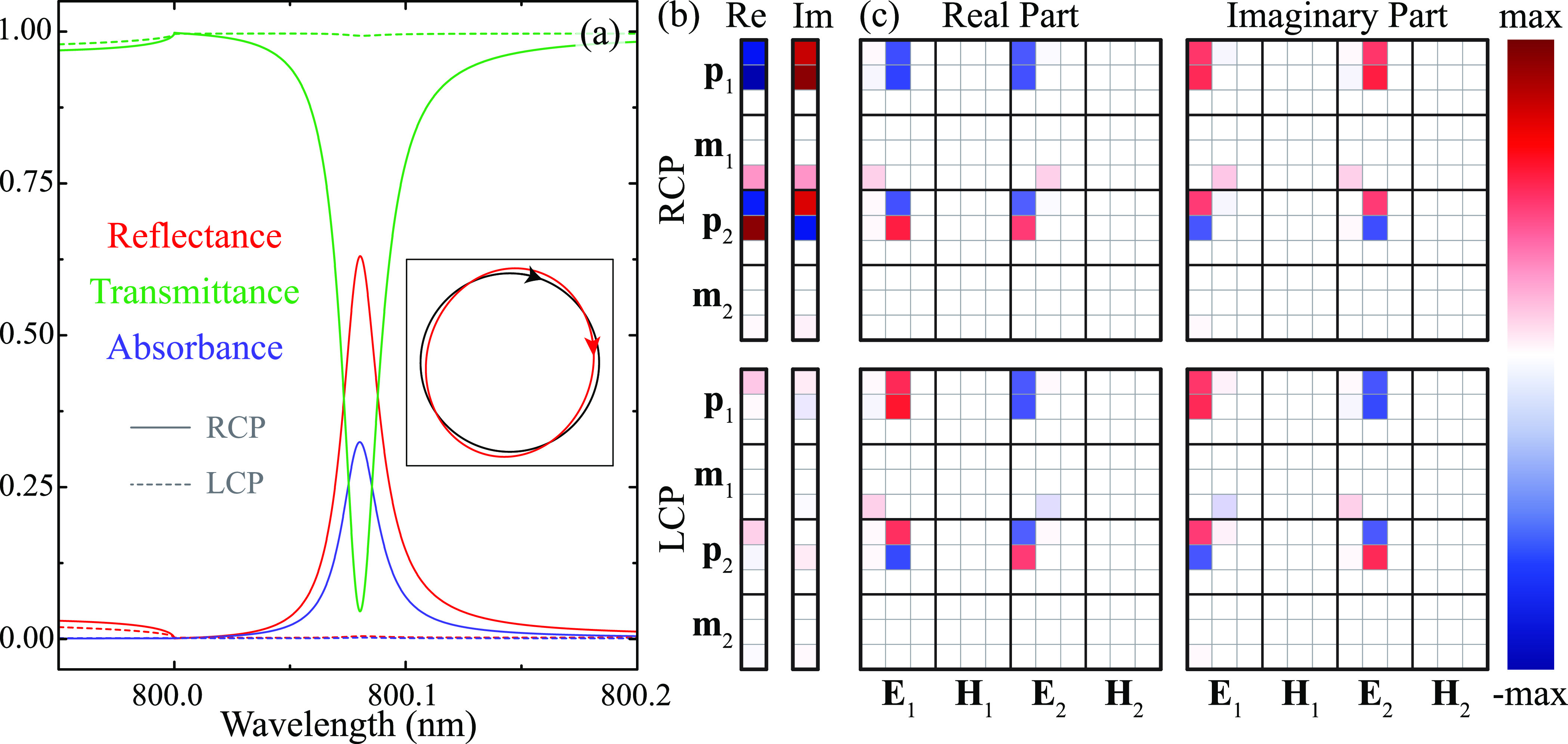
Analysis of the origin of the chiral response. (a) Reflectance, absorbance, and transmittance spectra of the 2.5-dimensional array with *a* = 800 nm, *D*_1_ = 120 nm, *D*_2_ = 190 nm, and **r**_2_ = (2*a*, 6*a*, 4*a*)/16. We use solid and dashed curves to indicate RCP and LCP excitation. The inset shows the polarization state of the incident (black curve) and reflected (red curve) field for RCP excitation, calculated at the lattice resonance wavelength (λ = 800.08 nm). (b) Real and imaginary parts of the electric and magnetic components of the dipole moments induced in the two particles of the unit cell at the lattice resonance wavelength. (c) Real and imaginary parts of the decomposition of the dipole moments of (b) (see main text). In both cases, the top (bottom) panels correspond to RCP (LCP) excitation. Notice that we normalize all of the quantities shown in panels (b) and (c) to the amplitude of the corresponding incident field, and, therefore, all of them have units of volume. The color bar is common for panels (b) and (c), and its maximum value is 4.1 × 10^6^ nm^3^.

So far, we have focused on describing the chiral lattice resonances supported by 2.5-dimensional bipartite arrays, but it is equally important to understand their physical origin. To that end, we center our attention on Array 3 with *z*_2_ = *a*/4, i.e., **r**_2_ = (2*a*, 6*a*, 4*a*)/16, since this system supports the strongest lattice resonance with the largest chiral response among the arrays analyzed. We plot the transmittance, reflectance, and absorbance spectra of this array in Figure 4(a), for both RCP (solid curves) and LCP (dashed curves) excitation. Examining these results, we observe that, for RCP excitation, the strong dip in the transmittance associated with the lattice resonance corresponds to a peak in both the reflectance and the absorbance, with values at resonance (λ = 800.08 nm) of *T* ≈ 0.05, *R* ≈ 0.63, and *A* ≈ 0.32, and a quality factor of 4 × 10^4^. In sharp contrast, the array shows an almost negligible response under LCP excitation, resulting in *T* ≈ 0.99 at that same wavelength. Therefore, this system behaves as a practically perfect circular dichroic filter, which blocks 95% of RCP light but transmits practically all LCP light. Moreover, it also acts as a chiral mirror that reflects only RCP light with 63% efficiency. Unlike conventional mirrors, which flip the handedness of circularly polarized light after reflection, chiral mirrors preserve it.^83^ To verify this behavior, we plot, in the inset of Figure 4(a), the polarization state of the incident (black curve) and reflected (red curve) field for RCP excitation. We calculate the polarization state at the wavelength of the lattice resonance taking into account the propagation direction. As anticipated, the field reflected by the array upon RCP excitation has the exact same polarization as the incident one, with only a slight change in ellipticity. Importantly, the same behavior can be obtained for LCP excitation by changing the sign of *z*_2_.

The chiral lattice resonance supported by the 2.5-dimensional array is the result of the collective interactions between its constituents. Therefore, we can get a deeper insight into their physical origin by looking at the dipole induced in the particles of the array. To that end, in Figure 4(b), we plot the electric and magnetic components of the dipoles induced in particles 1 and 2 of the array considered in Figure 4(a) at the wavelength of the lattice resonance. As one might have expected, the induced dipoles are much stronger for RCP (top panels) than for LCP (bottom panels) excitation. Furthermore, the main contributions to these dipoles are the *x* and *y* components for the electric dipoles and the *z* component for the magnetic ones. Since we are always considering normal incidence, the excitation of a magnetic dipole along the *z*-axis is a direct consequence of the interaction between the particles. Comparing the magnitude of the electric and magnetic dipoles, we observe that the latter are significantly smaller. Despite this, the magnetic response of the particles plays a crucial role. As shown in Figure S2 of the Supporting Information, if the magnetic response of the particles is neglected, the chirality of the lattice resonance completely disappears.

To understand how the interactions between the particles result in these induced dipoles, we can dissect the different contributions that give rise to them. As explained in Equation 2 of the Methods, the dipoles induced in the particles **d**_*i*_ = (**p**_*i*_, **m**_*i*_) are the result of , where  is the *i*,*j*-th block of the effective polarizability of the array , and **F**_*j*_ = (**E**_*j*_, **H**_*j*_) are the components of the incident electromagnetic field acting on particle *j* of the unit cell. Notice that, since we use Gaussian units, **p**_*i*_ and **m**_*i*_ have the same units, as do **E**_*j*_ and **H**_*j*_. In Figure 4(c), we plot the real and imaginary parts of each of the components resulting from the element-wise multiplication of each row of  with **F**. The top and bottom panels display the results for RCP and LCP excitation, respectively. The sum of the elements in each row results in the component of the induced dipole located immediately to their left. Interestingly, both polarizations excite the exact same components, namely, the *xx*, *xy*, *yx*, and *yy* for the electric dipoles, and *zx* and *zy* for the magnetic ones. The crucial difference is that, while, for RCP excitation, the terms with *i* = *j* have the same sign as those with *i* ≠ *j*, and hence add up constructively, for LCP excitation, these terms have opposite signs, thus canceling each other. Therefore, based on these results, we can conclude that the origin of the chiral lattice resonances supported by 2.5-dimensional bipartite arrays is the constructive and destructive interference between the electric and magnetic dipoles induced in the two particles of the unit cell.

**Figure 5:**
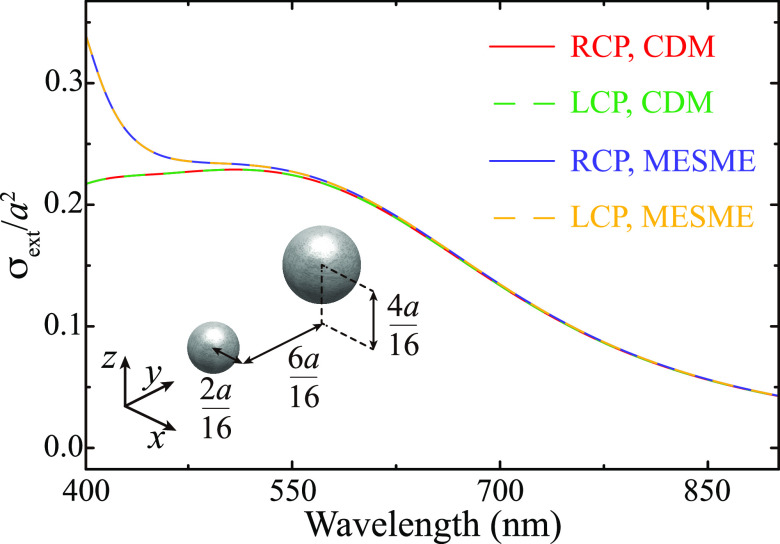
Achirality test for the unit cell. Extinction cross-section σ_ext_ of an individual unit cell of the 2.5-dimensional array considered in Figure 4 calculated with the CDM. The geometry of the particle dimer is described by the inset schematics. The system is excited with a plane wave propagating along the *z*-axis with either RCP (red solid curves) or LCP (green dashed curves) polarization. For comparison, the blue solid and yellow dashed curves show σ_ext_ obtained from a full numerical solution of Maxwell’s equation using the MESME approach.

Although we have already justified the achirality of the unit cell of the arrays under consideration using symmetry arguments, for the sake of completeness, we provide a direct proof of it in Figure 5. To that end, we calculate the extinction cross-section σ_ext_ of an individual unit cell of the array considered in Figure 4. The pair of particles, whose spatial arrangement is described by the inset schematics, is excited with a plane wave propagating along the *z*-axis. The red solid curve shows the results calculated with the CDM for RCP excitation, which are exactly identical to those obtained for LCP excitation (green dashed curve). To ensure that there is not a chiral response arising from higher-order modes not captured by the dipole approximation, we also plot, with blue solid and yellow dashed curves, the value of σ_ext_ obtained from a full numerical solution of Maxwell’s equations using a multiple elastic scattering of multipole expansions (MESME) approach.^84,85^ These spectra show no chiral response and also demonstrate that the accuracy of the CDM is excellent for wavelengths λ ≥ 550 nm, which includes the spectral range considered in this work. Below 550 nm, the contribution of the quadrupole modes of the particles becomes more significant.

**Figure 6:**
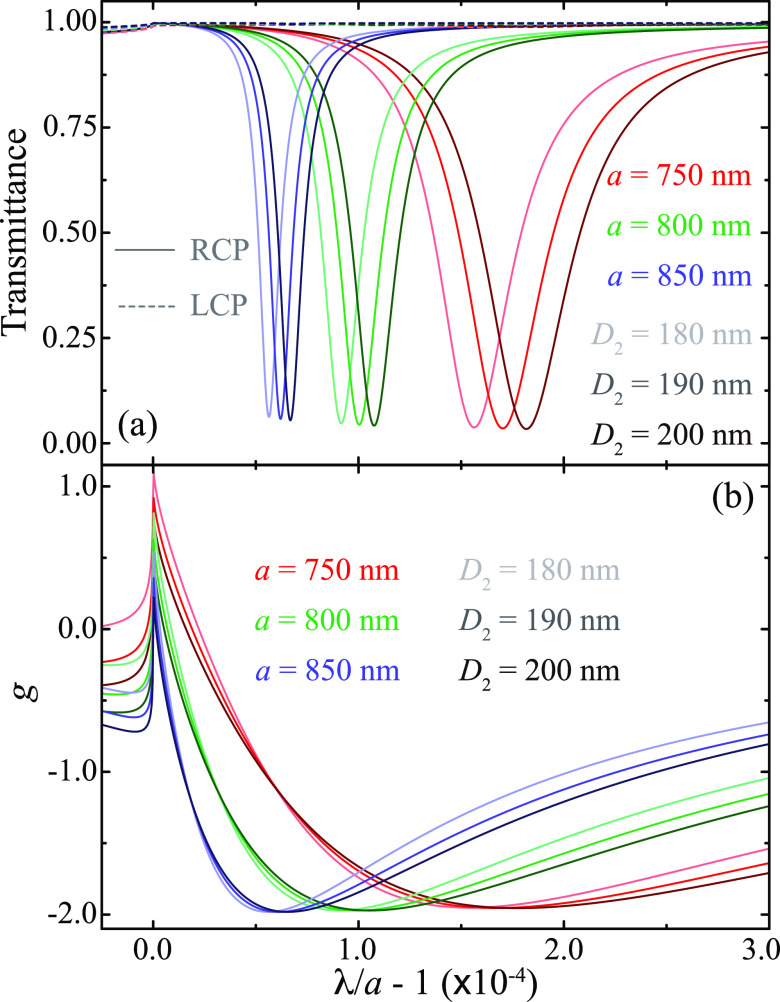
Effect of the array period and particle size on the chiral response of the array. (a) Transmittance for arrays with different values of *a* (see color code) and *D*_2_ (see shade code) under RCP (solid curves) and LCP (dashed curves) excitation. In all cases, *D*_1_ = 120 nm and **r**_2_ = (2*a*, 6*a*, 4*a*)/16. (b) Dissymmetry factor for the arrays analyzed in panel (a).

Up until now, we have analyzed square arrays with period *a* = 800 nm built from particles with diameters *D*_1_ = 120 nm and *D*_2_ = 190 nm. To prove that all of the results described in this work are general and not just a consequence of the particular choice of parameters, we perform an analysis of the response of arrays with different values of *a* and *D*_2_ in Figure 6. In particular, as indicated by the legend, Figure 6(a) shows the transmittance spectra for arrays with *a* = 750–850 nm and *D*_2_ = 180–200 nm, under RCP (solid curves) and LCP (dashed curves) excitation. In all cases, *D*_1_ = 120 nm and **r**_2_ = (2*a*, 6*a*, 4*a*)/16. Analyzing these results, we observe that the increase in the period or the decrease in the diameter of particle 2 results in the lattice resonance moving closer to the Rayleigh anomaly and narrowing its lineshape. These behaviors, which have been described in previous works,^9,76^ are associated with the increase of the collective nature of the lattice resonance.^17,30^ A similar trend is observed for the dissymmetry factor displayed in Figure 6(b). Moreover, the increase in the ratio *a*/*D*_2_ leads to larger values of *g*, practically reaching the theoretical limit of −2. For instance, *g* ∼ −1.98 for the array with *a* = 850 nm and *D*_2_ = 180 nm. Therefore, these results confirm that the behavior described in this work is general, thus establishing a mechanism to achieve chiral lattice resonances based on the use of 2.5-dimensional arrays.

## Conclusions

In summary, we have explored the optical response of 2.5-dimensional bipartite arrays made from the combination of a two-dimensional square lattice with a three-dimensional achiral unit cell. Using a rigorous approach based on the coupled dipole model, we have shown that these systems can sustain lattice resonances with almost perfect chiral behavior. The necessary conditions to obtain such resonances are (i) the use of two particles of different sizes and (ii) the placement of one of them in a low-symmetry position outside of the plane of the array. To illustrate this approach, we have analyzed the response of an array of period *a* = 800 nm, made of a unit cell with two silver particles of diameters *D*_1_ = 120 nm and *D*_2_ = 190 nm placed at positions **r**_1_ = (0, 0, 0) and **r**_2_ = (2*a*, 6*a*, 4*a*)/16. Under RCP excitation, this system supports a strong chiral lattice resonance, which results in *T* ≈ 0.05, *R* ≈ 0.63, and *A* ≈ 0.32 at the resonance peak, with a quality factor of 4 × 10^4^. In contrast, it displays a negligible response for LCP excitation, with *T* ≈ 0.99. In consequence, the array behaves as a nearly perfect circular dichroic filter and a chiral mirror, which preserves the handedness of the reflected light. Importantly, the chiral response of the arrays considered here does not arise from the response of individual constituents, as is the case for arrays made of chiral structures, but rather from their collective interaction. Indeed, by decomposing the dipole moments induced in the particles, we have shown that the chiral response is the result of the constructive and destructive interference between the electric and magnetic dipoles induced in the two particles composing the unit cell.

For simplicity, we have assumed that the 2.5-dimensional arrays are surrounded by vacuum, but our analysis can be readily extended to any other homogeneous dielectric environment, with the only expected change being a shift in the spectral position of the lattice resonances. However, it is well known that any asymmetry between the media above and below the array can hinder the lattice resonances and, hence, potentially affect the chiral responses predicted here. Fortunately, prior works have studied this problem and proposed strategies to minimize its effects,^86^ including the use of index-matching fluids.^87^ On the other hand, the presence of an inhomogeneous environment helps to break the mirror symmetry with respect to the array plane, potentially leading to an increase of the chiral response of the arrays.^59^

The fabrication of this type of arrays is within reach of current experimental technology, as demonstrated by recent works.^88−90^ For instance, few-layer three-dimensional plasmonic arrays have been obtained by self-assembly of silica-coated gold nanoparticles that, once infiltrated with an index-matching fluid, behave as arrays suspended in a homogeneous environment.^88^ Moreover, arrays with unit cells made of metallic nanostructures with different heights, and located at different planes, have been fabricated using an approach based on the binary-pore anodic aluminum oxide template technique.^90^

The results of this work provide a strong theoretical understanding of the chiral response of the lattice resonances supported by structurally achiral 2.5-dimensional arrays, and therefore are expected to have far-reaching implications from an application point of view. For example, the dissymmetry in absorbance displayed by these systems upon excitation with light of different handedness could be exploited in thermoplasmonics applications^30^ as a mechanism to control the heating of the array. Similarly, invoking reciprocity arguments, this same dissymmetry could be utilized to implement arrays emitting thermal radiation with nonvanishing optical helicity,^91^ or, if supplemented with a gain medium, sources of chiral light.^92^

## Methods

### Coupled Dipole Model

In order to describe the optical response of a 2.5-dimensional array built from a unit cell containing *N* particles, we use the coupled dipole model (CDM).^4,5,9,78−80^ This approach is justified when the particles are significantly smaller than both the wavelength of light and the period of the array. Within the CDM, and using Gaussian units, we model each of the particles as a point dipole **d**_*i*,μ_ = (**p**_*i*,μ_, **m**_*i*,μ_) with both electric **p**_*i*,μ_ and magnetic **m**_*i*,μ_ components. Furthermore, we use Greek indices to denote the unit cell to which the particle belongs and Latin ones to label the different particles within a unit cell. Accordingly, the position of any given particle in the array is specified by the sum of the vector **T**_μ_, which indicates the location of the unit cell μ, and **r**_*i*_, which determines the position of particle *i* within the unit cell. It is important to note that, while **T**_μ_ is contained in the *xy*-plane, **r**_*i*_ is a fully three-dimensional vector. The particles of the array are excited by a plane wave electromagnetic field **F**_*i*,μ_ = (**E**_*i*,μ_, **H**_*i*,μ_) with wavevector **k** and wavenumber *k* = 2π/λ. The components of the field are given by  and , with **e**_0_ and **h**_0_ being, respectively, the unit vectors of the electric and magnetic field components, and **k**_∥_ the projection of **k** onto the *xy*-plane. The dipole induced in an arbitrary particle of the array satisfies
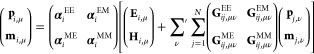
1where EE, EM, ME, and MM stand for the electric–electric, electric–magnetic, magnetic–electric, and magnetic–magnetic components, respectively, and the prime in the first summation indicates that the terms ν = μ are excluded from it when *i* = *j* because a dipole does not interact with itself. Furthermore, **α**_*i*_^ς^ represent the different components of the polarizability tensor of the particles, while **G**_*ij*,*μν*_^ς^ are the components of the dipole–dipole interaction tensor. The latter are defined as
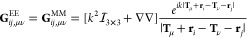
and
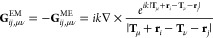
with  being the 3 × 3 identity matrix. Exploiting the periodicity of the array, we can seek solutions of Equation 1 in the form of Bloch waves . By doing so, we get
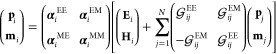
where  represent the Fourier transform of the different components of the dipole–dipole interaction tensor, usually referred to as the lattice sum. The equation above can be solved as
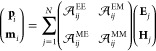
with  being the ς component of the *i*,*j*-th block of the 6*N* × 6*N* tensor  that contains the effective polarizability of the array. This quantity encodes the optical response of the array, which stems from the interplay between the individual response of the particles described by **α** and their mutual interactions determined by the lattice sum .

We can particularize the result shown above for the bipartite arrays considered in this work:
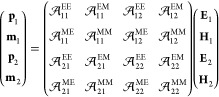
2Notice that, since we consider spherical particles made of silver, **α**_*i*_^EM^ = **α**_*i*_^ME^ = 0, while **α**_*i*_^EE^ and **α**_*i*_^MM^ are diagonal and can be computed from the dipolar Mie scattering coefficient^85^ (see Figure S3 of the Supporting Information).

Once the induced electric and magnetic dipoles are known, we can calculate the electric field created by the array at an arbitrary point **r** = (**R**, *z*) as **E**_rad_ = *∑*_**q**_**E**_**q**_, where **q** are the reciprocal lattice vectors, and
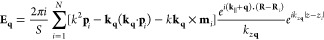
where *S* is the area of the unit cell, **r**_*i*_ = (**R**_*i*_, *z*_*i*_) are the positions of the particles in the unit cell, **k**_**q**_ = (**k**_∥_ + **q**, ± *k*_*z***q**_), and . Notice that the upper (lower) signs apply for *z* larger (smaller) than *z*_*i*_. Finally, we calculate the transmittance, reflectance, and absorbance of the array from the power transmitted, reflected, and absorbed, respectively, normalized to the incident power.

## Supporting Information Available

Benchmark of the coupled dipole model against full numerical solutions of Maxwell’s equations. Analysis of the role of the magnetic response in the chirality of the arrays. Plot of the electric and magnetic polarizabilities of the nanoparticles. (PDF)
