# Supplementary material for: Chiral Lattice Resonances in 2.5-Dimensional Periodic Arrays with Achiral Unit Cells
Source: ACS Photonics. 2023 May 10;10(6):1925–35. doi: 10.1021/acsphotonics.3c00369 (PMC10288824; doi:10.1021/acsphotonics.3c00369)
Supplement: Supplementary file 1 — ph3c00369_si_001.pdf [file ph3c00369_si_001.pdf]

Supporting Information: Chiral Lattice Resonances in 2.5-Dimensional Periodic Arrays with Achiral Unit Cells

Luis Cerdán,† Lauren Zundel,‡ and Alejandro Manjavacas*,†,‡

†*Instituto de Óptica (IO-CSIC), Consejo Superior de Investigaciones Científicas, 28006 Madrid, Spain*

‡*Department of Physics and Astronomy, University of New Mexico, Albuquerque, New Mexico 87106, United States*

E-mail: a.manjavacas@csic.es

Table of Contents

- Figure S1 .... Page S2
- Figure S2 .... Page S3
- Figure S3 .... Page S3

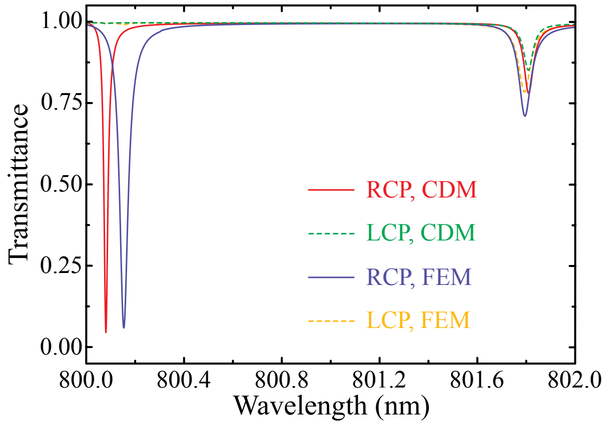

Figure S1: Benchmark of the coupled dipole model (CDM) against full numerical solutions of Maxwell's equations obtained using a finite element method (FEM) solver. As indicated by the legend, the red solid and green dashed curves correspond to the results obtained using the CDM, while the blue solid and yellow dashed curves show the results from the FEM simulation. In all cases, we use solid and dashed curves to represent RCP and LCP excitation, respectively. Furthermore, the period of the array is $a = 800\,\text{nm}$, the diameters of the nanoparticles are $D_1 = 120\,\text{nm}$ and $D_2 = 190\,\text{nm}$, and the position of particle 2 is $\mathbf{r}_2 = (2a, 6a, 4a)/16$. Clearly, the agreement between the CDM and FEM is excellent, with the lower-wavelength resonance being only slightly shifted and the higher-wavelength one having a slight change in strength. Importantly, the dissymmetry of the responses to RCP and LCP excitation remains identical in the FEM results.

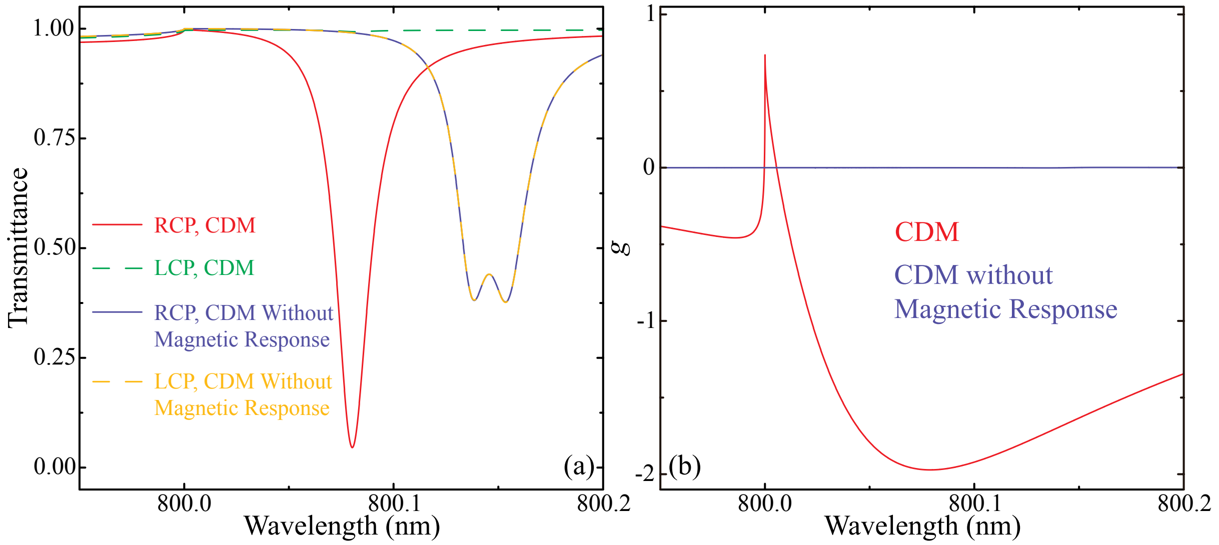

Figure S2: Analysis of the role of the magnetic response in the chirality of the 2.5-dimensional arrays. (a) Transmittance for an array with period $a = 800\,\mathrm{nm}$, particle diameters $D_1 = 120\,\mathrm{nm}$ and $D_2 = 190\,\mathrm{nm}$, and $\mathbf{r}_2 = (2a, 6a, 4a)/16$. As indicated by the legend, the red solid and green dashed curves correspond to the results obtained using the full CDM described in the manuscript, while the blue solid and yellow dashed curves correspond to results computed with the same method but neglecting the magnetic response of the particles. In both cases, we use solid and dashed curves to represent the results for RCP and LCP excitation, respectively. (b) Dissymmetry factor for the results obtained with (red curve) and without (blue curve) the magnetic response of the particles. Interestingly, by neglecting the magnetic response, the chiral behavior of the lattice resonance completely disappears.

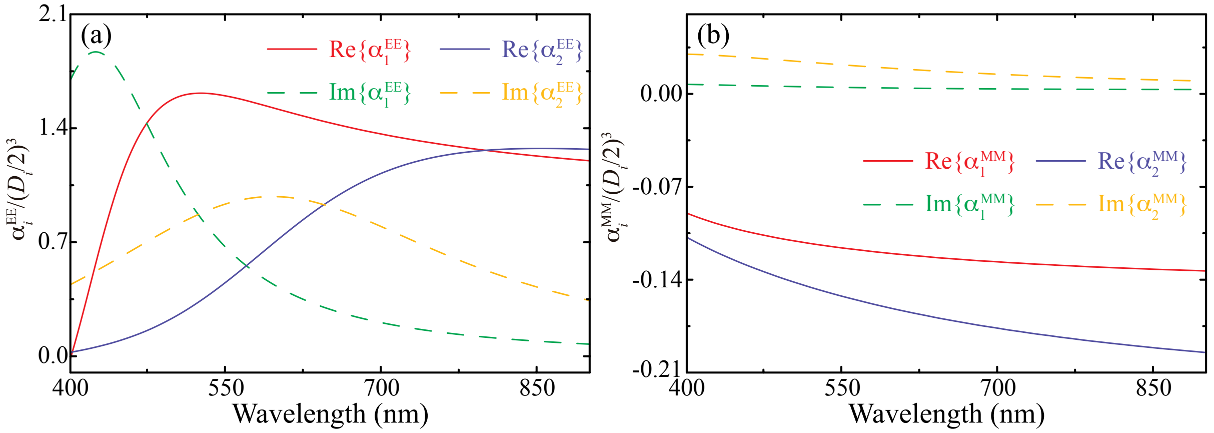

Figure S3: Nanoparticle polarizability. Electric α_i^{EE} (a) and magnetic α_i^{MM} (b) polarizabilities for nanoparticles with $D_1 = 120\,\mathrm{nm}$ (red and green curves) or $D_2 = 190\,\mathrm{nm}$ (blue and yellow curves). As indicated by the legend, solid and dashed curves correspond to the real and imaginary parts of the polarizabilities, respectively. In all cases, the polarizability is normalized to $(D_i/2)^3$.
